# Event and Comment

**Published:** 1921-02

**Authors:** 


### DENTAL REGISTER

THE MAGAZINE OF DENTISTRY

Vol. LXXV FEBRUARY, 1921 No. 2

### EVENT AND COMMENT

**Prosthetic Technique** Doctor Leon Williams in a statement in the *Journal of the National Dental Association*, of December, 1920, makes a statement that is quite true as a generalization, but needs considerable modification to make it applicable. He says: "The highest accomplishment in the teaching of dental prosthesis is not being followed in practice in more than one or two, if in any, dental college in the country. It would seem that for all colleges a simplified technique could be made that all would teach."

The difficulty seems to be that we have not yet achieved a complete standard of technique that can be demonstrated, or that any considerable number of teachers can adopt. It would be as impracticable to adopt a definite prosthetic technique as it would be to adopt a uniform method of operative technique, or of surgery, or even of violin playing. We have had many books and articles on the technique of prosthetic dentistry and good ones too; but as yet the perfect book is to be yet written. This subject can not as yet be stated as a science, it is as yet only an art, and will necessarily be subjected to the rules controlling in art rather than in the more scientific forms. To state the rules of prosthetics that will cover the art without harmonizing with the laws of physics and mechanics is not as simple a problem as it would seem.

Our practices have been to depend upon our technical art as we know and practice it, and to call for help largely when forced to act only when the law serves us best; what we mean is that we obey laws which suit us, and disregard such as do not seem essential. In short there are so many principles in practice that may control in science but seemingly they operate only in the realm of our art in practice. Consequently there are very few rules of practice that are standardized either by science or in art. If one were to examine critically our theories we should be forced to say that dentistry has utilized science when it has pleased and art when it has been convenient. As a consequence our theories of practice are not yet standardized, nor are they likely to be so very soon.

It is difficult to see how we can ever hope to have any standards of practice in prosthetic art so long as men are free to adopt man-made theories of the sciences and arts of practice. Since that is the present situation we must be content with theories that are only artistic in name and lacking in reality. We don't like to have Dr. Williams or anyone else tell us that we have no scientific, or artistic basis for a real artistic form of teaching or practice. In fact, it is not quite so bad as that seems; for we know that we have done much to utilize the art feature of our practice, and many of the theories we know because they have been tried out. Our trouble is wholly due to the fact that we have grown so fast in methods of service that we have overlooked the biology of our sciences. The facts of life have not changed, but as we have learned them they are continually new and we are fast getting acquainted with the newer facts. We shall need to grow with the science of evolution until we shall come to understand what is true before we can hope to standardize even so simple an art as prosthetic techniques.

